# On logicality and natural logic

**DOI:** 10.1007/s11050-021-09184-0

**Published:** 2021-07-14

**Authors:** Salvatore Pistoia-Reda, Luca San Mauro

**Affiliations:** 1 Departament de Traducció i Ciències del Llenguatge, Universitat Pompeu Fabra, Barcelona, Spain; 2 Institute of Discrete Mathematics and Geometry, Vienna University of Technology, Vienna, Austria

**Keywords:** Language logicality, Triviality, Ungrammaticality, Acceptable analyticities, Functional vs. nonfunctional vocabulary, Meaning modulation

## Abstract

In this paper we focus on the logicality of language, i.e. the idea that the language system contains a deductive device to exclude analytic constructions. Puzzling evidence for the logicality of language comes from acceptable contradictions and tautologies. The standard response in the literature involves assuming that the language system only accesses analyticities that are due to skeletons as opposed to standard logical forms. In this paper we submit evidence in support of alternative accounts of logicality, which reject the stipulation of a natural logic and assume instead the meaning modulation of nonlogical terms.

## Language logicality

The ‘logicality of language’ hypothesis refers to the idea that the language system contains a deductive device to exclude analyticities – that is, contradictions and tautologies (Gajewski 2002, 2009; Chierchia 2013, 2021; Del Pinal 2019, 2021; Pistoia-Reda and Sauerland 2021). Standard evidence in favor of the logicality of language comes from the distribution of exceptive phrases, *there*-existentials, and negative polarity constructions (von Fintel 1993; Gajewski 2008; Barwise and Cooper 1981; Chierchia 2013; Abrusán 2014). Structures like (1), (2), and (3) are said to be excluded from the language on account of their obligatory association with logically trivial contents.

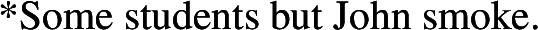

(2)

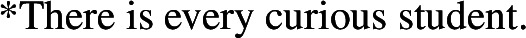

(3)

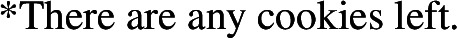

Let us focus on exceptives. The construction in (1) is said to be associated with the contradictory content reported below in (4b). In essence, the contradiction emerges from a conflict between the exceptive (“but”) and the existential quantifier (“some”). For illustration, let us denote by student and smoke, respectively, the collection of all students and all smokers, and by the constant *j* the individual John. Then, on the one hand, we have that the exceptive should make the quantified formula “some students smoke” false on student but true with respect to a relevant subset *X* of student, e.g. student ∖{*j*}; yet, on the other hand, we know that the existential quantifier is an *upward monotone* quantifier, which implies that if the quantified formula is true over *X*, then it should be true over all supersets of *X*, including student. Hence, according to the analysis we have a contradiction, and (4b) provides the logical reason why the language system excludes (1), i.e. why (1) is not an acceptable sentence.

(4)

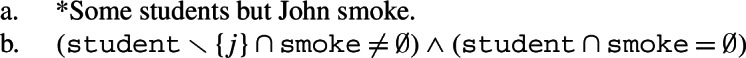

It is important to note that this account of exceptives is motivated by the systematic acceptability of sentences like (5a) and (6a), in which exceptives interact with downward monotone quantifiers. In the context of universal quantification, exceptive constructions are associated with clearly informative contents, as is illustrated in (5b) and in (6b).

(5)

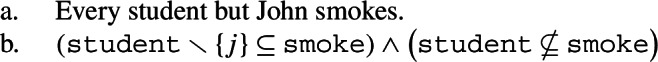

(6)

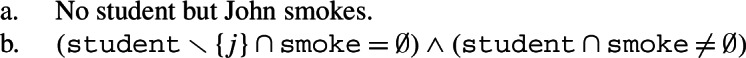

## Language logicality based on skeletons

Crucially problematic evidence for the logicality of language hypothesis comes from the observation that acceptable analyticities do occur. This is illustrated by the familiar contradictions and tautologies reported in (7)-(10). As a consequence of this puzzling evidence, the literature generally assumes that the logicality of language hypothesis cannot hold unrestricted. More precisely, special properties of the deductive device are assumed to be needed to explain why some, though not all, analyticities are excluded from natural language.

(7)

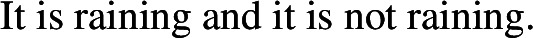

(8)

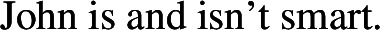

(9)

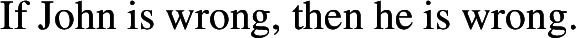

(10)

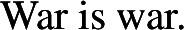

The standard response to the problematic evidence (sometimes referred to as the *analyticity puzzle*) involves assuming that logicality operates not on standard logical forms but rather on so-called logical skeletons (Gajewski 2002, 2009; Chierchia 2013). The latter are very austere and abstract representations that result from the nonuniform substitution of lexical items. The central idea of the account is that ungrammaticality is observed if and only if logical skeletons are analytic (see discussions on L-analyticity and G-triviality in Gajewski 2002, §3.3 and Chierchia 2013, pp. 49-54). Now, when analyticity is due to the co-occurrence of two or more lexical items, as in grammatical analyticities, this procedure ensures that the corresponding logical skeleton will not be analytic. When, on the other hand, analyticity would be preserved for any choice of lexical items, as in ungrammatical structures, the logical skeleton will be analytic. This is illustrated by the analytic skeleton associated with exceptives in (11b) and the informative skeleton associated with familiar acceptable contradictions in (12b).

(11)

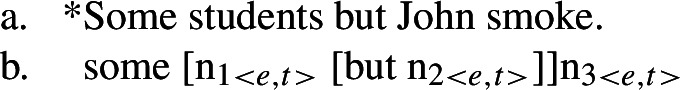

(12)

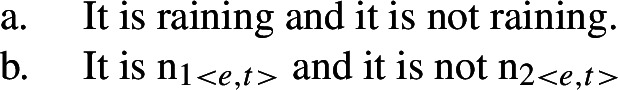

Although it is in line with familiar results on permutation invariance (van Benthem 1989), the skeleton proposal raises philosophical concerns. To realize this, consider the obvious implication of this proposal that logicality requires an intrinsically *linguistic* logic, which would be essentially different from classical systems. Indeed, if one assumes logicality to operate on skeletons, one is then forced to accept that most if not all inferential schemes and logical principles are not valid for logicality, since these are for the most part based on dependencies between nonlogical terms. Granted, this observation in itself does not constitute an argument against the skeleton proposal, and to strengthen the case against it one should additionally provide philosophical considerations against the very possibility of a natural logic; we would be sympathetic to such attempt. However, as discussed by Del Pinal (2019, pp. 21-24) (see also Abrusán 2019, pp. 340-341), there are versions of the skeleton proposal (e.g. Chierchia’s influential account of negative polarity constructions) that appeal to classical principles such as the law of non-contradiction to account for the triviality of ungrammatical structures such as (3), which makes one wonder whether the idea of a linguistic logic is ultimately tenable for those cases.

## Against language logicality based on skeletons

In a similar vein, in the present note we submit a novel argument showing that the assumption of a linguistic logic is not obligatory for those accepting logicality. From a philosophical perspective, the essence of the argument is that one can invoke logicality without “trepidation” (Gajewski 2002, p. 4) since a substantive characterization of the deductive system is not necessary. In particular, what we would like to show is that the same logical principle that justifies the analytic status of the ungrammatical exceptive construction (1) also justifies the analytic status of a suitably derived variant of (1). The crucial observation is that this variant is clearly grammatical. We understand this observation to suggest that logicality isn’t necessarily paired with a peculiar (or “exotic”; Abrusán 2019, p. 342, Del Pinal 2021, p. 23) deductive device.

In order to present the logic of our argument, let us begin with the analysis of one of the acceptable sentences with exceptives in universal quantification, namely (5a). For illustration, let us denote by *P* the unary predicate of smoking; obviously we have that *x* ∈ smoke if and only *P*(*x*) holds. In order to derive the relevant variant of (5a), we assume a very general equivalence between conjunction and universal quantification, which we describe in (13). We take it that the formula in (14b) is an adequate variant of (5a). In particular, what this variant expresses is that any collection of students such that they all smoke must necessarily exclude John. This, according to our understanding, captures the content of (5b) fairly accurately. In addition, we submit that (14a) provides one possible natural language translation of the logical variant. For concreteness, in order to translate (14b) into natural language, we are assuming that the relevant domain *D* consists of three students (say Alice, Bob, and John). Note that this assumption is made for the mere sake of simplicity, since the cardinality of *D* should not affect any grammaticality issue. Under this assumption, then, our translation appears straightforward.

(13)

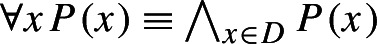

(14)

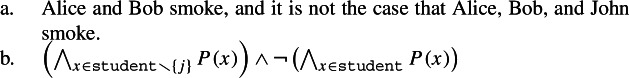

Let us now turn to the analysis of the ungrammatical construction with exceptives in existential quantification, namely (1). In this case, we submit that the formula in (16b) constitutes an adequate propositional variant of the representation in (4b), which provided the logical reason for the unacceptability of (1). This formula again derives from (4b) with the assumption that existential quantification is equivalent to generalized disjunction. Let us also consider a natural language translation of this variant, sentence (16a), where we assume again that |*D*| = 3. Our take is that the sentence so construed is acceptable – certainly less deviant than standard examples with exceptives and existentials such as (1).

(15)

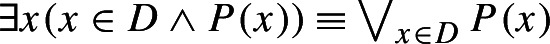

(16)

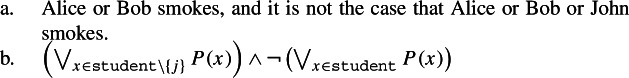

As is easy to check, the logical variant now under consideration crucially amounts to a contradiction. Our observation is that the source of this contradiction is the same as that of the contradiction in the original formula in (4b). We would like to emphasize that the analogy between (16b) and (4b) is, according to our understanding, not a mere superficial resemblance. In fact, it is a principle of classical logic that the disjunction satisfies the following weakening principle, which is importantly analogous to the upward monotonicity of the existential quantifier: *If* *ϕ* *holds, then* *ϕ*∨*ψ* *holds for any choice of* *ψ*. More generally, if a disjunction holds (or equivalently, if an existential formula is true over a certain domain), there is no way to make it false by adding other disjuncts (equivalently, by expanding the domain). We conclude that it cannot possibly be the case that a logical system that realizes the analytic status of (4b) does not also realize the analytic status of (16b) (we suspect that this conclusion is also valid for the other cases). The difference in grammaticality between (1) and (16a) is more plausibly related to the possibility of making the latter sentence informative via modulation of its nonlogical material (in line with the account of Del Pinal 2019, 2021; see also Pistoia-Reda and Sauerland 2021 for a possible extension). Assuming different representations is pointless if one doesn’t have a different logic underlyingly.

To make the latter suggestion more precise, consider a context in which the speaker wants to convey that adding John to the relevant group of people actually changes something with respect to what counts as smoking. This makes the content associated with the sentence informative: it prevents the contradiction via modulation of the meaning of the predicate. Note that this possibility immediately follows, for instance, from Del Pinal’s more pragmatic version of the logicality approach. According to this account, when an analytic interpretation is due to a dependency between nonlogical terms, a reinterpretation strategy, i.e. *rescale* or ‘ℜ’ in symbols, can apply to modulate the meaning of at least one of the occurrences of the predicate, thus removing the analyticity. Such a strategy is easily shown to be ineffective when the analytic interpretation is actually due to the functional vocabulary, like in the case of (1), and we have a single occurrence of the predicate. On the other hand, if we focus on our variant, it is easy to realize that the strategy is indeed sufficient to remove the contradiction that is literally expressed in that case. The contradiction would be removed, for instance, by specifying (with $\{x : \mathfrak{R}^{+}_{c} (P(x)) \}\subseteq \{ x : P(x)\}$) the meaning of the second occurrence of the predicate. But then the acceptability of our natural language translation (16a) is clearly expected.

(17)

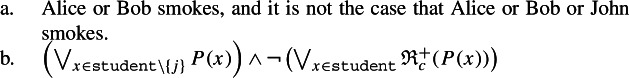

## Concluding remarks

Let us conclude. Based on unacceptable analyticities, the logicality of language hypothesis assumes a strict interaction between logic and grammar, to the point that some constructions are excluded from the language on account of their logical status. However, since acceptable contradictions and tautologies do occur, the hypothesis is standardly paired with the assumption that the deductive device only detects L-analyticities, i.e. analyticities arising at the level of logical skeletons as opposed to standard logical forms. In recent discussion, concerns have been raised as to the “exotic” nature of the resulting deductive device, in which few if any classical inferential schemes and logical principles apply. In this note, we provided a novel argument showing that the assumption of an intrinsically linguistic logic is not obligatory for those accepting logicality. In particular, we submitted that the same logical principle justifying the analytic status of certain ungrammatical structures, such as those involving exceptives in existential quantification, also justifies the analytic status of suitably derived variants. The crucial observation is that such variants are in fact grammatical.
